# 143 National strategy of physical activity in Georgia - process and results supporting sustainable development

**DOI:** 10.1093/eurpub/ckae114.019

**Published:** 2024-09-26

**Authors:** Kakha Gvinianidze

**Affiliations:** WHO, Tbilisi, Georgia

## Abstract

**Purpose:**

National strategy for promoting physical activity (PA) in Georgia aims to decrease insufficient PA in whole population, including children and adults. It is comprehensive document addressing key issues related with physical inactivity and covering all relevant sectors on central and municipal levels. Currently draft of the document is submitted to the government for official approval.

The document considers specific activities for the groups of population, such as pregnant women, children and youth, adults, people with disabilities and elderlies.

**Project description:**

Process has been initiated in 2021 by creating multisectoral group of PA under the National Center for Disease Control and Public Health with support of WHO and funding of EU. The group consists of state, municipal and nonstate representatives of health, education, sport, urban planning, transport and environment. First step was wide scale desk review and situation analysis. This was followed by brainstormings, drafting of the policy document and large-scale policy dialogue with diverse sectors.

Draft of the National Strategy and Action Plan of PA 2024-2030 includes improving PA programs and other opportunities in kindergartens and schools, workplaces, universities; services and opportunities for pregnant women, elderlies, people with disabilities; strengthening active mobility, tourism; increasing scale and attractiveness of green spaces, access to sport infrastructure and programs; integration of PA in health services; building institutional capacity, systematization of surveillance and national communication campaign.

**Conclusion:**

As a practical result of this work it is expected Georgia to have comprehensive national policy for health promoting physical activity with all associated benefits for health and economy of the country.

Nature of comprehensive PA policy requires relevant sustainable development elements to be widely considered in reforms of diverse sectors and therefore document development process includes large scale dialogue. Accordingly, PA on policy development stage became interconnecting issue and platform for policy dialogues regarding specific sustainable development issue of diverse sectors and it is expected to be continued and strengthened in course of its implementation. It may add to the experience and knowledge both from PA and sustainable development discourses especially for developing societies.

